# Supplementary material for: Cooperative pathways for the green transformation of heavily polluting enterprises: A four-party game-driven mechanism for green M&A
Source: PLoS One. 2025 May 7;20(5):e0322411. doi: 10.1371/journal.pone.0322411 (PMC12058201; doi:10.1371/journal.pone.0322411)
Supplement: Attachment — Submitted filename: Response to Reviewers.docx [file pone.0322411.s002.docx]

**Response to Reviewers**

In response to the reviewer’s comments, we have revised the manuscript and provide our responses to the reviewer’s suggestions as follows.

Dear Dr. Manuel Herrador,

Thank you for your constructive feedback on our manuscript, which helped us recognize the shortcomings in the simulation implementation section. Based on your suggestions, we have made every effort to provide a detailed description of our data sources and have listed the references we consulted. The revisions in the manuscript can be found in the section "5. Simulation of systems in different contexts", where the newly added content is highlighted in red.

Additionally, in this response, we have provided a more thorough analysis of our data sources. You may particularly focus on our reply to point seven from the first reviewer’s comments. Incorporating both your recommendations and the reviewer’s suggestions, we have made appropriate modifications to the content and have referenced additional literature to further strengthen the manuscript’s persuasiveness.

Thank you again for your valuable input. We will do our utmost to complete the revisions and improve the manuscript.

Best regards

Authors

**Reviewer #1:**

**Review Comment 1:**

**1.This paper explores the behavioral game theory between four stakeholders: government, media, heavily polluting enterprises, and the public. It is a relatively rigorous management science paper.**

We sincerely thank you for your thorough evaluation and feedback on our paper. We highly value your input, especially your recognition of the rigor and significance of our research. In this study, we constructed a four-party evolutionary game model to analyze the strategic interactions among governments, media, heavily polluting enterprises, and the public. We also examined the dynamic evolution of their behaviors under different policy environments. Through our model analysis and numerical simulations, we aim to provide theoretical support for improving green governance policies. We will address your specific comments carefully and revise the manuscript accordingly to enhance its academic contribution and practical relevance. Furthermore, we have recoded the references used in our responses to make them more accessible to reviewers. The reference codes in the revised manuscript may differ from those in the review comments, and we kindly ask for your understanding.

Please note, as we made adjustments to the manuscript during revision, we introduced new references to support the persuasiveness of the article. In the References section, we have highlighted the newly added references in red.

**Review Comment 2:**

**2.The introduction section can be expanded to include the research motivations and potential marginal contributions, and a comparison with previous studies should be analyzed and discussed.**

We sincerely appreciate the reviewers’ feedback, which helped us identify shortcomings in the introduction section. Based on your suggestions, we have further refined the introduction to expand on the research motivation and potential marginal contributions. We have also incorporated an analysis and discussion of previous studies. The specific revisions are as follows:

Regional economic and social development has not only accelerated global industrialization and modernization but also placed immense pressure on environmental resources and ecosystems. The unexpected outbreak of the COVID-19 pandemic and frequent extreme climate events have further disrupted global economic and social stability, forcing nations to reassess their development models(1). In response, many countries and regions have strengthened public health systems(2, 3), promoted green energy and sustainable development strategies, and leveraged technological innovation to improve crisis response capabilities(4). These measures have helped mitigate some of the adverse effects.

At the same time, the overlapping impacts of environmental issues have driven governments worldwide to prioritize the coordinated development of the economy, society, and environment as a critical future growth strategy. In China, the government has elevated ecological civilization to a national strategy. By improving environmental policy tools and implementing carbon neutrality goals, the government has made scientific and systematic environmental regulation a key means to achieving sustainable development(5, 6).

Against this backdrop, the green transformation and upgrading of heavily polluting enterprises have become a focal point of policy and public attention. As environmental regulations tighten, these enterprises face increasing pressure to transform. However, they also receive policy incentives to reduce their environmental impact through green mergers and acquisitions (M&A), technological innovation, and other means(7-9). Balancing regulatory constraints with incentives, while achieving green transformation, has emerged as a pressing issue for both academia and industry. Successful transformations not only contribute to ecological civilization but also provide valuable lessons and pathways for achieving harmonious economic and environmental development.

Green transformation is increasingly recognized as a key pathway for China’s high-quality economic growth(10). In the context of escalating global climate change and environmental crises, the sixth United Nations Environment Assembly emphasized the need for the international community to jointly address climate change, restore natural ecosystems, and strive for a pollution-free future. These goals have set a clear direction for global enterprises pursuing green development.

Green mergers and acquisitions, as a vital approach to driving green transformation, offer distinct advantages over traditional internal environmental investments and green innovation. Green M&A typically involves shorter cycles, lower risks, and faster integration of environmental resources and technological advantages(11, 12). By adopting a green development philosophy in target selection, deal structuring, and post-merger integration, green M&A enhances corporate energy efficiency and emissions reduction. It also sends a positive “carbon reduction” signal to the market, contributing to sustainable development goals(13). For heavily polluting enterprises, green M&A represents a key pathway for achieving deeper environmental transformation and serves as a core policy tool for green resource allocation under China’s ecological civilization and carbon neutrality objectives(14). Consequently, green M&A has become a preferred strategy for advancing sustainable development, improving competitiveness, and supporting high-quality growth, making it a central focus of academic and practical research (15).

During the green transformation process, heavily polluting enterprises typically face two strategic options: On one hand, they can pursue green M&A to achieve resource integration, technological innovation, and brand enhancement, thereby securing long-term government subsidies and competitive market advantages. On the other hand, some may adopt short-term greenwashing strategies to evade regulation, lower costs, and reap short-term gains(16). However, if greenwashing is truthfully reported by the media, it can lead to reputational risks, loss of market share, and potentially severe government penalties. Clearly, this strategic decision is not merely a matter of internal cost-benefit analysis; it is shaped by external factors such as government regulation, media oversight, and public feedback, all of which interact dynamically.

Existing studies on corporate green transformation tend to take a single-dimensional perspective. Some research focuses on the incentives and penalties of government regulatory policies. For example, Cranston (1982) discussed the fundamental structure of regulatory systems and their application in environmental governance(17), while McKean (1980) examined enforcement costs and difficulties under stringent regulations(18). These studies highlight the importance of external constraints imposed by government policy tools on corporate behavior. However, focusing solely on government perspectives fails to explain why firms continue to engage in greenwashing under real-world conditions. Corporate strategic choices depend not only on government policies but also on market information and social oversight. Other research examines the role of media in transmitting environmental information and monitoring firms. Bulger and Davison (2018) emphasized the media’s positive role in enhancing corporate transparency and shaping public environmental awareness(19), while Caled and Silva (2022) explored the risks of misinformation in digital media and its impact on corporate behavior(20). This body of literature demonstrates that media can expose corporate misconduct through truthful reporting. However, media may also collude with firms for financial gain, producing false reports and undermining external oversight. Additionally, some scholars have studied the public’s role in environmental governance, highlighting how public environmental awareness(21) and information discernment can promote corporate environmental responsibility and exert external pressure on greenwashing behavior(22, 23). Nevertheless, research that focuses solely on public oversight often overlooks the interaction effects between public supervision, government regulation, and media reporting.

While these studies offer valuable insights, they often fail to treat government, firms, media, and the public as an integrated system. Consequently, they struggle to reveal the complex interaction mechanisms and strategy evolution processes among these stakeholders. In reality, corporate decision-making during green transformation results from the combined influence of government policies, media information dissemination, and public supervision. Only a comprehensive model that captures the dynamic feedback effects and adjustment processes among multiple stakeholders can provide a more accurate depiction of the green transformation landscape.

To address this theoretical gap, this study employs evolutionary game theory to develop a four-party game model involving government regulators, heavily polluting enterprises, media, and the public. The model assumes that all participants exhibit bounded rationality and approach optimal equilibrium solutions through iterative games under incomplete information conditions(24, 25). It incorporates key variables such as government regulation (strict versus lenient), corporate strategies (green M&A versus greenwashing), media coverage (truthful versus false reporting), and public oversight (information discernment and feedback). By doing so, the model dynamically depicts the interactions and strategic adjustments among these stakeholders.

In the four-party game model constructed in this study, the strategic choices of each stakeholder stem from an internal cost-benefit tradeoff during the green transformation process. For heavily polluting enterprises, the decision often comes down to choosing between green mergers and acquisitions (M&A) and greenwashing strategies. Opting for green M&A allows these enterprises to integrate green resources, promote technological innovation, and potentially gain government subsidies and market recognition, thereby establishing a long-term competitive advantage(8). In contrast, while greenwashing may yield short-term economic benefits, it carries significant risks of reputational damage, market sanctions, and legal consequences once exposed by the media(16). Therefore, an enterprise’s strategic decisions depend not only on internal cost-benefit considerations but also on external factors such as policies, regulatory pressures, and market feedback.

Second, the primary motivation of government regulators lies in improving environmental quality, promoting green industry development, and increasing revenues from environmental protection taxes and fines, thereby enhancing public health and social welfare(17, 18). However, governments must also contend with high administrative costs and enforcement risks. As a result, they carefully balance strict and lenient regulation in order to maximize long-term environmental benefits and maintain social stability.

Media, as both an information disseminator and a key social watchdog, must balance its pursuit of commercial revenue with the need to maintain credibility. Truthful reporting on corporate misconduct can enhance the media’s reputation and foster public trust(19), yet it may also lead to a loss of advertising and sponsorship income from those corporations. On the other hand, false reporting can generate short-term financial gains, but under strict government regulation and increased public scrutiny, it exposes the media to administrative penalties and reputational damage(20).

Finally, as the ultimate beneficiaries of environmental governance, the public is motivated to obtain accurate information and engage in oversight to ensure that companies fulfill their environmental responsibilities. Although this may involve certain time and effort costs, the public’s objective evaluation of corporate environmental performance can help prevent greenwashing and encourage stricter government regulation(26). Conversely, if the public blindly trusts the media or encounters opaque information channels, their supervision over polluting enterprises may weaken, potentially increasing environmental risks.

This study makes several contributions. First, it advances theoretical innovation. Prior research largely focused on individual stakeholders or bilateral relationships, such as the effects of government regulation on corporate behavior(17) or the role of media in information transmission(19). This study, by constructing a four-party game model, integrates the interactions among government, firms, media, and the public into a unified framework. It reveals how these stakeholders continuously adjust their strategies over repeated games under conditions of limited information and rationality, ultimately achieving an overall equilibrium. This integrated approach provides new theoretical insights into the driving mechanisms behind green transformation (8, 16). Second, it introduces methodological innovation. By combining evolutionary game theory with replicator dynamics analysis, this study systematically describes the learning and adaptation processes of each stakeholder in a dynamic environment. Unlike traditional static models, this approach captures the strategic evolution trajectories that emerge as stakeholders receive feedback and update their information, thus offering a more accurate representation of the complexities involved in green transformation(19, 20). Additionally, this study employs both quantitative and qualitative methods, providing a theoretical and methodological foundation for subsequent empirical research. Lastly, it offers practical implications. Although this study uses China as a case for simulation analysis, the game model’s fundamental logic—corporate cost-benefit tradeoffs, government regulation strategies, media oversight effects, and public feedback mechanisms—has universal applicability. For example, in regions like the European Union and North America, strict environmental regulations, carbon trading systems, and independent media supervision also play critical roles in promoting corporate green transformation. With minor adjustments to model parameters, this framework can guide green transformation efforts in other countries or regions, offering theoretical and policy recommendations(27).

In conclusion, this study’s multi-stakeholder game model not only provides a theoretical explanation for the strategic choices involved in the green transformation of heavily polluting enterprises but also reveals the complex interactive mechanisms among external factors such as government regulation, media oversight, and public feedback. The findings contribute to the game theory literature on green transformation and environmental governance while offering practical policy insights for harmonizing diverse interests and achieving sustainable development across various contexts.

This paper is structured as follows: Section 2 outlines the problem and constructs the game model; Section 3 analyzes the stability of the stakeholders; Section 4 evaluates the stability of strategy combinations; Section 5 conducts simulations under different scenarios; Section 6 discusses the study’s implications and future research directions; Section 7 summarizes the conclusions.

**Revised Version Description:**

We have presented the modifications in response to the reviewer’s comments and made corresponding changes in the manuscript, highlighted in red. Please refer specifically to Section 1: Introduction.

**Review Comment 3:**

**3.What exactly does "the cost of strict regulation for the government" and "the basic revenue gained by the government from lenient regulation" refer to?**

We sincerely thank the reviewer for the valuable comments, which have helped us gain a more complete understanding of the shortcomings in our parameter definitions. In this paper, the government faces a trade-off between two different costs and benefits when choosing regulatory strategies. The core concepts are explained based on our model assumptions and related literature:

**(1) Costs of Strict Regulation:**

These costs primarily refer to the resources and expenses incurred by the government when implementing a strict regulatory strategy, including:

1)Administrative and enforcement costs: Strict regulation requires increased monitoring, inspections, and enforcement efforts by the government. This entails significant human, material, and time resources, as well as direct expenses for monitoring equipment, data collection, and processing(28).

2) Economic and social costs: Strict regulation can impose short-term burdens on businesses, potentially reducing economic vitality. Additionally, high regulatory intensity may provoke dissatisfaction among businesses and certain social groups, negatively affecting the government’s public image and political support(17).

**(2) Basic Benefits of Lenient Regulation:**

Compared to strict regulation, a lenient regulatory strategy can offer certain short-term benefits, such as:

1) Cost savings: Lenient regulation involves lower resource allocation and administrative costs, reducing public expenditure(18).

2) Economic and social effects: More lenient policies can lower compliance costs for businesses, stimulate economic activity, and promote growth. This can result in increased tax revenue and improved employment, aligning with short-term interests of certain businesses and communities. This approach may enhance government support in the short term. However, excessive leniency over the long term can lead to deteriorating market conditions and declining environmental quality, ultimately causing public dissatisfaction—a factor we have reflected in the “government leniency loss” parameter(17).

Incorporating these two parameters into the multi-stakeholder game model allows us to analyze how the government’s decisions regarding corporate green transformation account not only for the long-term benefits of environmental improvement, but also the various costs and benefits arising from different regulatory approaches in the short term. This provides a more comprehensive explanation of both green M&A and greenwashing behaviors.

**Revisions in the Updated Version:**

We have addressed the reviewer’s concerns by elaborating on these parameters in our response, and we have also made additional clarifications in the manuscript. The newly added and revised content is marked in red in the revised manuscript.

**Review Comment 4:**

**4.While it is understandable for enterprises to accept regulation, why would "the media" also "accept regulation"? The logic in Figure 1 should be reconsidered—why would the media accept regulation?**

Thank you for raising the question, which allows us to clarify why media should also be subject to regulation.

In the four-party game model presented in this study, media play a dual role: they serve as conduits for information and as overseers of corporate behavior and public opinion. This is detailed in **Fig. 1: Multi-subject game logic diagram.** While enterprises are regulated to prevent them from exploiting information asymmetries for greenwashing, media, if left entirely unchecked, may also act against the public good. For instance, financial incentives—such as accepting corporate bribes or prioritizing commercial partnerships—could lead to the dissemination of false information. This, in turn, could conceal corporate misconduct, erode public trust in environmental governance, and compromise the integrity of the entire system. The need to regulate media can be explained through three key points:

(1) Ensuring Information Transparency and Objectivity:

Government oversight helps maintain the transparency and reliability of information. Media outlets are critical channels for societal oversight, as their reports directly influence public perceptions of corporate environmental behavior. Without some degree of constraint, media may succumb to economic interests or political pressures, resulting in biased or inaccurate reporting. Such deviations not only enable corporate greenwashing but also mislead public judgment, thereby undermining the effectiveness of environmental governance mechanisms. As highlighted in the book *Media Capture: How Money, Digital Platforms, and Governments Control the News*, media are often influenced by capital and power, which necessitates external oversight mechanisms to preserve their independence and credibility.

(2) Safeguarding Public Interest and Social Justice:

In a multi-stakeholder game, media not only disseminate information but also act as guardians of public interest. By implementing appropriate regulatory measures—such as penalties for false reporting and encouragement of self-regulation and industry standards—governments can incentivize media to remain objective in their coverage of corporate activities. This reduces the risk of public misinformation and social inequities caused by inaccurate or misleading reports(19). Consequently, the public can access more accurate information, allowing them to more effectively monitor corporate behavior and support genuine green mergers and acquisitions.

(3) Preventing Media Capture:

Without effective regulation, media are vulnerable to “capture” by corporations or other interest groups. The aforementioned book on media capture notes that in the absence of regulation, media can lose their independence and oversight functions. Such a situation not only allows corporations to manipulate public perception through controlled narratives but also creates a vicious cycle that diminishes the effectiveness of both government and public oversight. Reasonable government regulation is therefore essential to prevent media capture, ensuring that media continue to function as legitimate and effective public watchdogs.

In summary, media regulation is not a simple restriction on free reporting. Instead, it aims to establish a fair and transparent environment for information exchange within the multi-stakeholder framework. By addressing potential biases and ensuring the media’s positive role in green transitions, regulation supports corporate adoption of genuine green M&A practices and promotes the coordinated development of environmental, economic, and social benefits.

**Review Comment 5:**

**5.Can the decision-making motives and influencing mechanisms of each stakeholder be further clarified to help readers better understand the interactions among stakeholders in the game model?**

Thank you very much for your feedback, which has provided us with a clearer understanding of the paper’s shortcomings. In response to the reviewer’s comments, we plan to refine the manuscript by addressing two key areas: First, we will add four separate paragraphs to the revised introduction to analyze in detail the core incentives and interaction logic of enterprises, governments, media, and the public in the context of the green transformation game. Second, we will expand the current model’s assumptions section to further clarify the parameter definitions, thus emphasizing the decision-making motivations and influence mechanisms of each stakeholder.

Given the word limit and our comprehensive responses to comment 2 (revised introduction) and comment 6 (revised model assumptions), we will present the relevant explanatory text added to the introduction as our response to this comment.

In the four-party game model constructed in this study, the strategic choices of each stakeholder stem from an internal cost-benefit tradeoff during the green transformation process. For heavily polluting enterprises, the decision often comes down to choosing between green mergers and acquisitions (M&A) and greenwashing strategies. Opting for green M&A allows these enterprises to integrate green resources, promote technological innovation, and potentially gain government subsidies and market recognition, thereby establishing a long-term competitive advantage(8). In contrast, while greenwashing may yield short-term economic benefits, it carries significant risks of reputational damage, market sanctions, and legal consequences once exposed by the media(16). Therefore, an enterprise’s strategic decisions depend not only on internal cost-benefit considerations but also on external factors such as policies, regulatory pressures, and market feedback.

Second, the primary motivation of government regulators lies in improving environmental quality, promoting green industry development, and increasing revenues from environmental protection taxes and fines, thereby enhancing public health and social welfare(17, 18). However, governments must also contend with high administrative costs and enforcement risks. As a result, they carefully balance strict and lenient regulation in order to maximize long-term environmental benefits and maintain social stability.

Media, as both an information disseminator and a key social watchdog, must balance its pursuit of commercial revenue with the need to maintain credibility. Truthful reporting on corporate misconduct can enhance the media’s reputation and foster public trust(19) (Bulger & Davison, 2018), yet it may also lead to a loss of advertising and sponsorship income from those corporations. On the other hand, false reporting can generate short-term financial gains, but under strict government regulation and increased public scrutiny, it exposes the media to administrative penalties and reputational damage(20).

Finally, as the ultimate beneficiaries of environmental governance, the public is motivated to obtain accurate information and engage in oversight to ensure that companies fulfill their environmental responsibilities. Although this may involve certain time and effort costs, the public’s objective evaluation of corporate environmental performance can help prevent greenwashing and encourage stricter government regulation(26). Conversely, if the public blindly trusts the media or encounters opaque information channels, their supervision over polluting enterprises may weaken, potentially increasing environmental risks.

**Revisions in the Updated Version:**

The additional and revised content in the manuscript’s introduction and model construction sections has been highlighted in blue.

**Review Comment 6:**

**6.The mechanism description in this paper is very rigorous, but there is a small flaw. The author is recommended to refer to https://doi.org/10.1016/j.esr.2024.101590 and** **https://doi.org/10.3390/agriculture15010023 to further enhance the content.**

Thank you very much for pointing out the weaknesses in our mechanism description and providing the relevant references for us to study. Your feedback has greatly improved our understanding of how to describe mechanisms more comprehensively in future papers. After analyzing and discussing the two references you provided, we realized that our paper’s mechanism description failed to adequately detail the decision-making motivations and influence mechanisms of each stakeholder. This aligns with your comment 5. Therefore, we have decided to rewrite the mechanism description and add the appropriate references to better clarify the motivations and influence mechanisms of all stakeholders.

Specific revisions are as follows:

**Hypothesis 1:** Participants in the Game.In this study, we selected government regulators, heavily polluting enterprises, media, and the public as the primary participants in the game model. This selection reflects the critical role these stakeholders play in environmental governance and green transformation. It is also strongly supported by evolutionary game theory and incomplete information game theory(24, 25). At the outset of the game, due to incomplete information and uncertainty about future economic trends, participants cannot accurately predict each other’s behaviors. As a result, they must approach equilibrium solutions through multiple rounds of iteration, continuously adjusting their strategies based on feedback to maximize their own benefits. For heavily polluting enterprises, the choice between green M&A and greenwashing revolves around the trade-off between long-term transformation benefits and short-term economic gains. Opting for green M&A can help integrate green resources and enhance technological innovation capabilities, while also securing government policy support and subsidies(16). Conversely, greenwashing may temporarily reduce costs and boost profits, but once exposed by truthful media reports, reputation risks and market share losses become substantial. Under conditions of bounded rationality, enterprises learn and adjust their strategies through iterative gameplay to maximize their overall benefits. The government’s primary goals include improving environmental quality, enhancing social fairness, and increasing public satisfaction, while also balancing regulatory costs and political risks. Strict regulation can yield environmental improvements and public support(17), but it also entails significant enforcement and administrative costs(18). Consequently, the government must weigh strict and lenient regulation, adjusting its strategies throughout the iterative game process to achieve long-term governance goals. Media, as a critical channel for information dissemination and oversight, must balance economic returns, reputational effects, and external regulatory pressures. Truthful reporting enhances media credibility and long-term brand value(19), but may forfeit short-term revenue from corporate partnerships. False reporting, on the other hand, may provide direct economic benefits (such as corporate bribes), but it risks administrative penalties and reputational damage under strict government regulation and heightened public information discernment(20). Media must adapt their reporting strategies based on external feedback to achieve a balance between economic and social returns in the iterative game. The public contributes to the game through information discernment and supervisory feedback. To accurately judge the authenticity of media reports, the public incurs certain time and effort costs. When the public detects corporate greenwashing, they may report these practices to the government, prompting further regulatory actions(19). In turn, public oversight pressures corporations and media to correct deviations, creating positive incentives throughout the iterative game process. With bounded rationality, the public gradually refines their information judgment skills, influencing corporate strategies and shaping government and media regulatory approaches.

**Hypothesis 2:** Strategy combination of the game. This paper focuses on the strategic choices made by the other three parties of the game when the heavy polluting enterprise chooses green mergers and acquisitions. So the strategy combinations in this paper are the heavily polluting enterprise (green mergers and acquisitions, greenwashing), the media (true reporting, false reporting), the government (strict regulation, loose regulation), and the public (objective identification, direct belief).

**Hypothesis 3:** The choice proportion of game strategies. The probability that a heavily polluting enterprise chooses green M&A is $e$ $(0\leq e\leq1)$, and the probability that it chooses greenwashing is $(1-e)$; the probability that the media chooses truthful reporting is $m$ $(0\leq m\leq1)$, and the probability that it chooses false reporting is $(1-m)$; the probability that the government chooses strict regulation is $g$ $(0\leq g\leq1)$, and the probability that it chooses lenient regulation is $(1-g)$; the probability that the public chooses objective identification is $p$ $(0\leq p\leq1)$, and the probability that it chooses to believe it directly is $(1-p)$.

**Hypothesis 4:**For heavily polluting enterprises, the choice between green mergers and acquisitions (M&A) and greenwashing strategies essentially involves weighing long-term strategic benefits against short-term gains. Opting for green M&A yields immediate benefits$Q_{s}$, but also incurs merger costs$Q_{h}$. Green M&A helps enterprises rapidly integrate green resources, enhance green innovation capabilities, and comply with strict government environmental regulations(8). Additionally, policies such as carbon trading systems provide further support through subsidies or tax incentives, increasing the economic returns of green M&A (29). These forms of government support are captured in the model as a policy subsidy parameter$Q_{c}$(30). In contrast, while greenwashing can deliver short-term gains$Q_{f}$, it involves certain costs$Q_{k}$, including internal governance expenses and potential legal risks. If the media truthfully report the greenwashing, the enterprise faces serious reputational damage and market penalties$Q_{d}$(16). To avoid these risks, some enterprises may bribe the media (bribe amount parameter$M_{s}$(31)) in an attempt to suppress the truth and temporarily evade scrutiny from the public and regulators(20, 32). Moreover, under strict government regulation, enterprises found engaging in greenwashing are subject to administrative penalties (penalty amount parameter$L_{s}$), further reducing their net profits. Thus, in making their strategic decisions, heavily polluting enterprises must balance the long-term technological and branding benefits of green M&A—along with positive policy incentives—against the reputational and market risks associated with greenwashing. This interplay of multiple external factors drives enterprises to rationally choose between green M&A and greenwashing strategies, ultimately aiming to maximize overall returns.

**Hypothesis 5:** In reporting corporate activities, media obtain a base revenue, denoted as$M_{w}$ which includes income from advertisements, subscription fees, and other business collaborations. When media choose truthful reporting, their objective and impartial reporting style can earn widespread social and public recognition, significantly enhancing their reputation value, denoted as$M_{c}$(33). This reputational gain helps build long-term brand trust and sustained collaborative relationships. However, exposing corporate greenwashing through truthful reporting may lead to reduced corporate partnerships or lower sponsorship amounts, causing a direct revenue loss, denoted as$Z_{s}$.On the other hand, if media opt for false reporting, they may receive additional short-term financial gains from corporate bribes, denoted as$M_{s}$(31).However, under strict government regulation, such behavior can result in administrative penalties, denoted as$L_{k}$(34).Moreover, when the public demonstrates strong information discernment and rationally identifies the media’s false reporting, the media can suffer further reputational damage, denoted as$L_{c}$(20). This negative impact not only weakens the media’s long-term competitiveness but may also prompt more stringent regulatory measures, further restricting their market share(32).

**Hypothesis 6:** When the government adopts a strict regulatory strategy, it receives basic revenue$A_{c}$, while also gaining added benefits$R_{c}$(33) from reduced environmental pollution, improved public health, and enhanced ecosystem quality. These positive outcomes not only encourage corporate green transformation but also generate long-term social feedback, bolstering government credibility and policy support(17). However, strict regulation incurs substantial enforcement costs$A_{s}$(28)associated with human resources, material inputs, and time. These costs represent a significant burden for the government. Faced with these ongoing expenses, the government may be inclined to choose a more lenient regulatory strategy, which can lower administrative expenditures and stimulate corporate economic activity in the short term. These short-term gains are denoted as$I_{p}$.Nonetheless, lenient regulation often leads to environmental degradation and negative public opinion regarding ineffective government oversight. This results in additional social and political losses, represented as$I_{n}$(17).As a result, the government must find a balance between the long-term environmental benefits of strict regulation and the short-term economic advantages of leniency. Achieving this balance involves weighing the direct costs and added benefits of each policy approach, while also considering the external social and market feedback’s impact on government credibility, thereby ensuring sustainable public governance.

**Hypothesis 7:** For the public, the positive effects of corporate green M&A include improved environmental quality, enhanced public health, and increased social welfare, collectively denoted as$G$(35).However, according to information asymmetry theory, the public must expend time and effort to verify the authenticity and completeness of media reports, incurring an identification cost$H_{c}$(19).If the public objectively identifies corporate greenwashing, they may, with a certain probability$\beta$(36), file complaints with the government and receive compensation$T_{g}$ from the companies involved, partially offsetting the negative impacts of environmental degradation. Conversely, as the relatively disadvantaged party in terms of information access, if the public blindly trusts the media’s false reports without conducting objective verification, they may suffer economic and environmental losses, denoted as$H_{s}$(37),due to misleading information. This cost-benefit tradeoff shows that while public supervision can encourage companies to adopt green transformation measures, it also entails significant costs and risks associated with information discernment.

**Revisions in the Updated Version:**

The additions and modifications have been highlighted in red in the manuscript. Regarding this particular issue, the reviewer may wish to focus on the section "2.2 Model construction".

**Review Comment 7:**

**7.Are the parameter values in the numerical analysis based on references from literature or real-world cases?**

Thank you for raising this question, which highlighted our oversight in specifying the sources of parameter assignments. In response, we have searched, analyzed, compiled, and calculated data from recent policy documents, relevant databases, reference literature, news reports, and real-world cases to provide a more robust data foundation. In the current version, we have also referred to the established parameter assignment practices published in PLOS ONE (38, 39). The specific basis for our parameter settings is as follows:

To facilitate calculation, this study sets the annual revenue of an enterprise at 100 units.

**(1) The direct benefits that heavily polluting enterprises gain from choosing green M&A:** After reviewing relevant literature, we found clear evidence that green mergers and acquisitions (M&A) have positive economic impacts on heavily polluting enterprises. According to Xu, Y., Wang, W., Gao, H., & Zhu, H. (2024), green M&A improves environmental performance and generates economic benefits (40). Similarly, empirical research by He, S., Wei, Y., & Li, W. (2024) shows that heavily polluting enterprises experience an average revenue increase of 15% to 17% following green M&A(41). Based on these data, we consider setting the direct revenue gained from green M&A at 16 units to be a reasonable representation of its role in enhancing corporate economic performance.

**(2)** **The costs that heavily polluting enterprises incur when choosing green M&A:** Meanwhile, undertaking green M&A requires a significant investment of capital and resources. Xu, Y., Wang, et al. (2024) indicate that green M&A can substantially deplete corporate resources, often necessitating the reallocation of other internal investments to support these initiatives. For heavily polluting enterprises, the cost of green M&A typically accounts for about 30% of their annual revenue. Accordingly, this study sets the cost of green M&A at 30 units(40).

**(3) Government subsidies for the heavily polluting enterprise:** According to the relevant guidelines from the China Carbon Neutrality Policy Documents, the government is actively promoting green and low-carbon transformation and has introduced various measures to encourage heavily polluting enterprises to undergo transformation and upgrading through green M&A. The Opinions of the State Council on Accelerating Carbon Peak and Carbon Neutrality (2021) clearly state that fiscal subsidies, tax incentives, and green financial support are to be used to motivate companies to accelerate their green transformation, reducing energy consumption and carbon emissions. For example, Shandong Province offered a 15% corporate income tax reduction to green mining demonstration projects, incentivizing environmental investments. Similarly, policies have stipulated that for enterprises actively pursuing green M&A and integrating environmental technologies, the government can provide subsidies equivalent to approximately 15% of the M&A costs. These measures help alleviate financial pressures, promote green technology innovation, and advance green industry development. Based on these policy foundations and empirical studies by relevant scholars (16), this model sets the government subsidy for heavily polluting enterprises conducting green M&A at 4.5 units, which equals 15% of the M&A costs. This figure aligns with current policy orientations and fiscal incentive levels.

**(4) The benefits that the heavily polluting enterprise gain from choosing greenwashing:** Enterprises engaging in greenwashing can achieve significant short-term gains. According to a Unilever survey, 33% of consumers prefer brands claiming environmental responsibility, suggesting that exaggerating environmental performance can attract more customers and boost sales. The Global Greenwashing Risk Report indicates that heavily polluting enterprises can gain 15–25% of their market revenue in the short term through greenwashing. In order to account for the short-term market benefits that greenwashing may bring, this study sets the direct revenue obtained by heavily polluting enterprises choosing greenwashing at 15 units.

**(5) The direct costs that the heavily polluting enterprise incur when choosing greenwashing:** The costs associated with greenwashing are primarily reflected in public relations and marketing expenses, which are much lower than actual environmental investments. Reports indicate that many companies spend more on promoting a green image than on genuine environmental initiatives. According to the Global Greenwashing Risk Report, greenwashing-related costs typically range from 5% to 10% of revenue. Based on this and the findings of Stefan, A., & Paul, L. (2008)(42), we estimate the direct cost of greenwashing to be 7 units. This is significantly less than the 30 units for green M&A costs, highlighting the comparative affordability of greenwashing versus substantive environmental actions.

**(6) The losses incurred by the heavily polluting enterprise due to truthful media reporting of their greenwashing practices:** When greenwashing behavior is exposed by the media, enterprises face reputational damage, market penalties, and various economic losses. Numerous real-world cases have shown that once environmental violations are made public, companies are often forced to halt production for rectification, invest heavily in pollution control, and suffer a loss of consumer trust and significant stock price declines. For example, the Chongqing Ecological Environment Bureau publicly disclosed that a company in Chongqing faced a major environmental incident due to illegal discharges, resulting in direct economic losses of nearly 10 million RMB, along with production disruptions and market share loss. Taking into account these combined losses—such as reduced sales, increased remediation costs, and adverse capital market reactions—existing research estimates that the overall economic loss from environmental issues being exposed amounts to approximately 8% of annual revenue when normalized to 100 units. Thus, in this model, we assign the value of the loss incurred from greenwashing exposure as 8 units(16, 43).

**(7) The amount of bribery given by the heavily polluting enterprise to the media:** Some companies resort to bribing the media to suppress negative environmental news. In the "21st Century Network" extortion case, an investigation by Caixin in 2015 revealed that between 2010 and 2014, the media outlet signed advertising contracts with hundreds of companies each year, effectively collecting hush money that totaled several billion RMB. On average, each company paid millions to prevent negative information from being exposed. Based on data from China’s environmental pollution surveys, in heavily polluting industries, the amount paid to bribe media typically constitutes a small proportion (around 5%–8%) of annual revenue. With the model setting annual revenue at 100 units, assigning a bribery cost of 6 units is reasonable. This value reflects the typical costs incurred by companies to conceal pollution and aligns with real-world case data(20, 44).

**(8) The penalty amount imposed by the government on the heavily polluting enterprise:** China’s environmental regulations impose heavy penalties on heavily polluting enterprises that engage in greenwashing. For instance, the Water Pollution Prevention and Control Law of the People’s Republic of China (revised in 2017) stipulates that companies found guilty of malicious pollution discharge or false environmental claims can face fines in the millions. In enforcement practice, the Chongqing Municipal Ecological Environment Bureau once fined a company 1 million RMB for severe violations. In more egregious cases, such as the 2018 subsidy fraud scandal involving King Long New Energy Vehicles, penalties reached nearly 800 million RMB according to CCTV News. Given the considerable variation in real-world fines, this model uses standardized parameters (based on an annual revenue of 100 units). Considering the typical severity of violations and the corresponding penalties, we assign a fine of 9 units to greenwashing enterprises. This value reflects the strict enforcement practices in China and aligns with the penalty levels reported in relevant regulatory documents.

**(9) The basic revenue gained by the media from reporting on corporate actions:** Media can boost circulation and increase audience engagement by reporting on corporate news, which in turn attracts more advertisers and raises overall ad revenue. According to the Annual Report on China’s Media Industry (2019), during periods of significant corporate news coverage, some mainstream media outlets experienced an average advertising revenue increase of about 8%–12%. Additionally, research by Sparks, Colin, et al. (2016) shows a strong positive correlation in the Chinese market between heightened media attention to corporate news and higher advertising revenue(45). After standardization, this positive effect equates to approximately 10 units of revenue.

**(10) The reputation gain acquired by the media:**When media truthfully disclose corporate environmental behavior (e.g., exposing greenwashing), they enhance their credibility with the public and build long-term brand value. According to Bulger and Davison (2018)(19), consistently accurate reporting helps establish and maintain public trust. Although this trust may be difficult to quantify in the short term, it contributes positively to the media’s brand over the long run. Additionally, Pew Research Center’s findings indicate that in environments of higher news transparency, the public’s trust in media is relatively higher. While the economic and brand-value improvements may develop gradually, their positive impact is significant(46). Based on these studies, we estimate the reputational benefit from accurate reporting to be 1 unit. This value reflects the incremental increase in public trust and the cumulative long-term brand value gained by adhering to truthful reporting.

**(11) The loss of cooperative revenue for the media from partnerships with heavily polluting enterprises:** When media outlets choose to expose corporate misconduct, they often refuse to engage in "cover-up" agreements, thereby losing commercial partnerships with those companies, such as advertising contracts and special collaborations. According to the *Annual Report on China’s Media Industry*, advertising revenues for some mainstream outlets declined by 4% to 8% year-over-year during periods of negative corporate news exposure. This reflects the significant impact that corporate sponsorships have on overall media revenue. Based on these figures, we conservatively estimate that the lost commercial partnership revenue due to truthful reporting is approximately 4 units, representing the opportunity cost media incur when fulfilling their watchdog responsibilities.

**(12) The amount of government penalties faced by the media for false reporting:** In China, there are well-established mechanisms for penalizing false media reports. According to the Notice from the General Administration of Press and Publication on Strengthening the Management of False News (2021), media outlets and responsible individuals face severe sanctions if they publish inaccurate reports. These measures range from public reprimands and temporary suspensions of publications to revocation of journalist credentials and media licenses. In more serious cases, criminal charges may apply—such as extortion or defamation—resulting in further legal consequences. These administrative and judicial penalties not only impose significant fines on media outlets but often force them into internal reorganization and operational adjustments, restricting their ability to conduct business. This ultimately results in long-term economic and reputational losses. For instance, under the Notice from the General Administration of Press and Publication on Strengthening the Management of False News (2021), the fines and rectification requirements imposed on certain media outlets in recent years have placed substantial financial pressure on them. Moreover, research by Jiang, Zhiqian, et al. (2020) indicates that the cost of penalties for false reporting—encompassing both direct fines and the costs of implementing corrections—represents a significant burden for the industry, negatively impacting long-term operations and credibility(47). Based on these data and examples, we assign a value of 6 units to the comprehensive penalty cost incurred by media for publishing false reports in this model. This figure reflects not only the direct monetary fines imposed by regulators but also the broader negative impact on media brands and long-term operations.

**(13) The reputational loss faced by the media:** When media outlets publish false news, their credibility is severely damaged. Once the public becomes aware of a media outlet covering up the truth or disseminating false information, trust can erode rapidly, significantly reducing the outlet’s brand value. According to Gentzkow, M., & Allcott, H. (2017), public trust in media has declined noticeably in many countries in recent years(48). Bulger, M., & Davison, P. (2018) also emphasize that once media are perceived as lacking authenticity, their long-term brand value can suffer substantial harm(19). Additionally, Silva, M. J., & Caled, D. (2021) point out that the reputational damage from false reporting not only affects immediate financial performance but also weakens the media’s influence and competitiveness in the long term(20). Drawing on these surveys and academic studies, we estimate the reputational loss from false reporting at 5 units. This reflects the audience attrition and long-term economic impact caused by diminished trust and reduced influence on public perception.

**(14) The basic revenue gained by the government from strict regulation:** Strict environmental regulation not only allows the government to collect fines from violating companies but also generates direct economic benefits through several channels:Firstly, enhanced regulation supports the growth of green industries and improves environmental quality, which in turn increases revenue from environmental protection taxes.Secondly, better environmental conditions reduce public expenses on pollution-related medical care and social governance, saving public funds.Thirdly, by promoting green transformation, the resulting green economic effects drive local economic growth(49). According to the 2023 China Environmental Statistics Yearbook and the 2020 Ministry of Ecology and Environment Report of China, in some regions, the combined benefits from increased environmental tax revenue, green industry growth, and savings on healthcare and social governance costs often amount to about 8% of regional economic indicators(50). Based on these figures, and under the assumption that annual corporate revenue is standardized at 100 units, we set the direct benefits (excluding fine revenues) from strict regulation at 8 units.

**(15) The additional revenue gained by the government from strict regulation:** In addition to direct economic benefits, strict environmental regulation also helps the government gain public support and long-term advantages. According to a 2021 public environmental awareness survey by the Beijing Municipal Ecology and Environment Bureau, approximately 70% of respondents stated they would actively report environmental violations or pollution. This indicates that in regions where regulatory channels are accessible and public awareness is high, strict environmental oversight aligns with public expectations, enhancing the government’s credibility and administrative image. Moreover, research by Stern (2000) shows that stringent environmental regulation effectively prevents ecological damage and leads to long-term improvements in public health and socio-economic outcomes(26). Specifically, strict regulation reduces pollution, lowers public health expenditures, and promotes sustainable development, generating substantial yet hard-to-quantify long-term societal benefits. Based on these findings and theoretical underpinnings, our model assigns an additional benefit of 1 unit to reflect the government’s improved reputation, public health improvements, and long-term welfare gains resulting from strict regulation. This valuation captures both the public’s strong demand for environmental governance and the positive long-term impact of strict regulation on government credibility and societal well-being.

**(16) The cost of strict regulation for the government:** Implementing strict environmental regulation involves significant administrative costs, including environmental monitoring, personnel enforcement, technical equipment procurement, and judicial processes. According to data from the 2023 China Environmental Statistics Yearbook, national public expenditures on energy conservation and environmental protection in 2023 amounted to approximately 540 billion RMB, with 7%–15% allocated to regulation and enforcement, monitoring, and related activities. For example, some local government reports indicate that expenditures on inspections, enforcement, and technical equipment were substantial but placed considerable pressure on overall budgets (see 2023 China Environmental Statistics Yearbook and related data from the National Bureau of Statistics). In addition, Wang, H. (2002) noted that while stricter environmental regulation brings long-term environmental benefits, its direct administrative costs remain a significant portion of the economic structure(51). Based on these data and studies, we assign a value of 7 units to represent the cost of stricter regulation in our model. This value reflects the actual government expenditure on inspections, enforcement, judicial processes, and personnel and equipment, and aligns with publicly reported figures on regulatory costs as a share of total investment.

**(17) The basic revenue gained by the government from lenient regulation:** Under lenient regulation, local governments often relax environmental restrictions to attract investment and stimulate short-term economic growth. This allows polluting enterprises to reduce production costs and expand output, thereby driving local GDP growth and increasing tax revenues. In fact, numerous studies and real-world examples demonstrate that some local governments, facing economic development pressures, deliberately ease environmental oversight to attract investment. For instance, Godlewska, M., & Pilewicz, T. (2022) note that in certain resource-dependent and industrial regions, local authorities relaxed environmental standards, resulting in notable increases in tax revenue and employment(52). Additionally, local government fiscal revenue improved as polluting enterprises scaled up their operations. The 2018 Ministry of Ecology and Environment Report and various local government performance assessment documents also show that, in pursuit of short-term economic gains, some regions adopted lenient regulatory strategies, lowering environmental standards but achieving higher tax revenues and enhanced economic activity. Drawing from these studies and real-world cases, we set the baseline revenue for governments under lenient regulation at 15 units in our model. This value reflects the combined economic benefits local governments derive from relaxed oversight—such as increased tax revenues, GDP growth, and employment—achieved by lowering corporate environmental costs.

**(18) The losses faced by the government from lenient regulation:** Relaxed environmental oversight can lead to significant environmental and social risks, resulting in both direct and indirect losses for governments. On one hand, lenient regulation often causes environmental quality to deteriorate, sparking public discontent and collective protests. According to the 2019 China Environmental Protest Incident Statistics Report, there are roughly 500 environmental protests daily nationwide, and the number is increasing. This highlights the heightened social stability risks in regions where regulatory channels are accessible and public awareness of rights is strong(53). On the other hand, as higher-level authorities intensify their environmental inspections, the State Council Notice on Strengthening Environmental Inspections mandates strict accountability for local officials failing to manage environmental issues. In recent years, multiple local officials have faced dismissal or disciplinary action due to environmental negligence, as publicly reported. Based on these data and examples, we assign a loss value of 3 units in the model to quantify the adverse impacts of relaxed regulation—such as environmental degradation, public complaints, and higher-level reprimands—on local governments.

**(19) The positive effect of a enterprise's green M&A on the public:** When enterprises implement green mergers and acquisitions (Green M&A), they can reduce pollutant emissions, which directly benefits public health and the environment. The World Bank (2016) estimated that the economic losses caused by environmental pollution in China account for approximately 5.8% of GDP, largely due to increased healthcare expenditures and lost labor productivity(54). By achieving cleaner production through Green M&A, enterprises can reduce the adverse impacts of pollution on public health, effectively saving society from incurring these costs. Based on this reasoning and supporting studies(55), we assign a value of 6 units to represent the positive impact on public welfare from green mergers—indicating that the benefits in terms of improved health, safety, and environmental quality roughly equate to a 6% improvement relative to the baseline.

**(20) The public's discernment cost:** Public verification of corporate environmental claims requires a certain amount of effort in terms of time and energy. However, in recent years, environmental information has become increasingly transparent as media and non-governmental organizations provide valuable supervisory tools. For instance, the Institute for Public Environmental Research (IPE) has established a corporate emissions database, while outlets such as Daily Economic News regularly publish reports like the "A-share Green Weekly" that publicly disclose firms’ environmental performance. These platforms significantly reduce the cost for the public to obtain accurate information(56, 57). Based on this enhanced transparency, we set the average cost for public objective verification of corporate environmental behavior at 1 unit, reflecting that individuals only need to expend a relatively small amount of effort to verify information.

**(21) The probability of the public filing complaints to the government:** With growing environmental awareness, public attention to and participation in environmental issues have steadily increased. According to the 2021 Public Environmental Awareness Survey Report by the Beijing Municipal Ecology and Environment Bureau, approximately 70% of respondents stated they would file complaints or reports when encountering pollution incidents, reflecting a 10-percentage-point increase over previous years. This indicates a strong public willingness to supervise environmental behavior in areas where regulatory channels are accessible and environmental rights awareness is high. However, nationally, the situation varies. In more remote regions, limited regulatory access, information difficulties, and lower public awareness lead to a complaint rate of only around 10%(58). Taking into account the significant regional disparities in development and regulatory conditions, we conservatively set the national average public complaint probability at 40% (0.4) in our model. This reflects higher complaint activity in developed areas, while also accounting for lower engagement in more remote regions. As such, this value is both reasonable and representative.

**(22) The compensation amount given by the heavily polluting enterprise to the public:** Under the provisions of China’s new Civil Code (2021), ecological and environmental damages are clearly incorporated into the civil liability framework. This requires polluting enterprises to compensate for public health and property losses caused by environmental pollution(59). In recent environmental public interest lawsuits, courts have frequently ordered enterprises to pay compensation amounts ranging from millions to tens of millions of RMB(60, 61). For instance, in a high-profile pollution case in Chongqing, the court imposed compensation of several tens of millions of RMB, underscoring the significant financial responsibility placed on enterprises after major environmental incidents. Based on these legal standards, actual case precedents, relevant industry surveys, and academic studies, we set the compensation amount payable by enterprises to the public or society at 9 units in our model. This figure aligns with the stringent requirements of the new Civil Code and corresponds to the compensation levels observed in real-world cases.

**(23) The loss incurred by the public due to misinformation:** Misleading green claims by corporations can result in both direct and indirect economic and health losses for the public. On one hand, consumers who are led to believe in these claims often pay a premium for so-called “green” products. Haws et al. (2014) report that consumers typically pay a green premium of 1% to 5%(62), while other studies show that average premiums in certain markets are around 1.7%(63). If the advertised green attributes turn out to be false, this premium becomes a direct economic loss for consumers. On the other hand, misleading claims can reduce public vigilance toward polluting companies, delaying calls for pollution control measures and increasing long-term health risks and environmental degradation. Research by Stern (2000) and Slovic (2000) indicates that distortions in public perception of environmental risks can significantly diminish support for effective environmental policies(26, 64). This cognitive bias can have lasting adverse effects on public health and environmental conditions. Based on these studies and market survey data, we quantify the public’s loss from misleading green claims at 2 units. This reflects a modest yet notable level of loss in terms of economic expenditure and health-related environmental consequences.

Lastly, we must acknowledge that the credibility of simulation-based studies is lower than that of empirical research. Although we have made every effort to measure parameters through direct or indirect means, certain limitations remain. In the "Research Limitations" section, we have noted these shortcomings for potential readers, and we hope that future research in this area can be supported by more extensive empirical data.

After processing the simulation data, the parameter settings in this paper are as follows:

$Q_{s}=16$,$Q_{h}=30$,$Q_{c}=4.5$,$Q_{f}=15$,$Q_{k}=7$,$Q_{d}=8$,$M_{s}=6$,$L_{s}=9$,$M_{w}=10$,$M_{c}=1$,$Z_{s}=4$,$L_{k}=6$,$L_{c}=5$,$A_{c}=8$,$R_{c}=1$,$A_{s}=7$,$I_{p}=15$,$I_{n}=3$,$G=6$,$H_{c}=1$,$\beta=0.4$,$T_{g}=9$,$H_{s}=2$

**Revised Version Notes:**

Additions and modifications have been marked in red in the manuscript. Reviewers may wish to focus on the simulation implementation section for details on these changes.

**Review Comment 8:**

**8.Is this game model applicable only to China, or can it also be effective in other countries or regions?**

Thank you for raising this question. In response, we provide a detailed explanation here, and we have added a discussion on the practical significance of this study in the revised manuscript’s introduction section. This highlights that our game model is applicable not only in China but also in other countries or regions.

Our game model is based on evolutionary game theory, with a core focus on analyzing how enterprises, governments, media, and the public optimize their interests through multiple interactions under conditions of bounded rationality. In theory, this multi-stakeholder game mechanism is universal and not limited to China. In our study, we chose China as a case primarily because its policy background and societal oversight mechanisms provide a representative example of green transformation and environmental governance.

For instance, the Chinese government implements stringent environmental regulations but also faces high enforcement costs. Media, on the other hand, may demonstrate bias due to economic interests, yet public oversight and government regulations often push them toward greater objectivity. Meanwhile, the public plays a pivotal role in both information access and monitoring corporate behavior. These factors collectively create a balanced, dynamically evolving game system.

However, the basic logic of this game mechanism—firms weighing costs and benefits in their green transformation, governments balancing strict and lenient regulation, media making trade-offs between economic gains and maintaining credibility, and the public engaging in information discernment and oversight—applies in other countries and regions as well. While the specific parameters, policy intensity, media landscape, and public awareness may vary from region to region, the fundamental interaction dynamics are highly applicable. For example, whether in Europe, North America, or other developing countries, regulatory systems and media operations may differ from those in China, but the pressures faced by firms undergoing green transformation, government priorities for environmental governance, and public expectations of corporate environmental performance are universal phenomena. Below, I will analyze the four stakeholders in detail:

**(1)Corporate Behavior:**

Existing literature shows that in both developed and developing nations, heavily polluting firms choose between green mergers and acquisitions (M&A) and greenwashing based on their evaluation of long-term transformation benefits (such as green resource integration, technological innovation, and government subsidies) versus short-term cost avoidance (including reputational risk and market share loss)(8, 16). This pattern is observable across multiple countries, differing only in parameters and institutional context.

**(2)Government Regulation:**

Regulatory mechanisms are not unique to China. The studies by Cranston (1982) and McKean (1980) discuss regulatory strategies from the perspectives of cost-benefit trade-offs and enforcement costs(17, 18). These theoretical foundations have been validated in studies conducted in Europe, North America, and other regions. For instance, the EU Emissions Trading System (EU ETS) operates on a combination of strict regulation and market incentives, demonstrating that the environmental improvements and cost-benefit analyses associated with stringent regulation have broad relevance(27).

**(3)Media Behavior:**

Media, as a key channel for information dissemination and oversight, operates under similar pressures worldwide. Bulger & Davison (2018) and Caled & Silva (2022) have shown that globally, media face a dual challenge: on one hand, pursuing economic gains, and on the other, maintaining credibility and social responsibility(19, 20). Whether in China, Europe, or North America, the trade-offs between "truthful reporting" and "false reporting" reflect similar motivations, providing theoretical support for our game model’s cross-national applicability. Additionally, Media capture: How money, digital platforms, and governments control the news (2021) discusses how media influence by capital and political power is a universal phenomenon(32).

**(4)Public Oversight:**

In monitoring corporate behavior, the public’s decisions are primarily driven by information costs and environmental awareness. Studies in multiple countries have verified the role of improved public information literacy and proactive oversight(19), indicating that the public’s influence and feedback mechanisms in environmental governance are universal. Public scrutiny of media reports and complaints about greenwashing, whether in China or other nations, are critical forces in driving corporate green transformation.

In summary，although our simulation study uses China as a case due to its representative policy background and social structure in green transformation and environmental regulation, the fundamental logic and interaction mechanisms of the game model are broadly applicable. With adjustments to the model’s parameters to account for institutional, cultural, and market differences, this multi-stakeholder game model can also explain green transformation challenges in other countries or regions.

**Revision Notes:**

Based on the reviewer’s comment, we have added an analysis of the practical significance of this study in Section 1 (Introduction). This analysis highlights the broad applicability of the game model constructed in this paper.

**Reviewer #2:**

**Review Comment 1:**

**You have done excellent work in the field of Game theory, especially in terms of the four agents. Generally, revision is necessary for the manuscript, however, this paper can be accepted now due to its high quality.**

We sincerely appreciate the reviewer’s high appraisal of our contributions to game theory and four-stakeholder analysis in this study. We are delighted to see that you regard our work as of high quality and have acknowledged its value. To live up to your commendation, we have made further necessary revisions to improve the content and presentation of the manuscript. Once again, we thank you for your valuable feedback and support!

All in all, we would like to thank the editorial team and reviewers for your recognition of our work and the valuable suggestions for improvement. Based on your feedback, we have revised the manuscript once again. We are sincerely grateful for the opportunity to gain new insights into our study. We are looking forward to hearing from you!

# References

[1]Martin F M, Sánchez J M, Wilkinson O. The economic impact of COVID-19 around the world. FRB St. Louis Working Paper, 2022 (2022-30).https://doi.org/10.20955/r.105.74-88

[2]World Health Organization. Operational framework for primary health care: transforming vision into action. 2020.

[3]Wen H, Deng W, Guo Q. The effects of the environmental protection tax law on heavily polluting firms in China. Plos one, 2021, 16(12): e0261342. https://doi.org/10.1371/journal.pone.0261342

[4]Drucker, Peter, and Joseph Maciariello. Innovation and Entrepreneurship. 1985. https://doi.org/10.4324/9781315747453

[5]Wang Y, Xu S, Meng X. Environmental protection tax and green innovation. Environmental Science and Pollution Research, 2023, 30(19): 56670-56686. https://doi.org/10.1007/s11356-023-26194-z

[6]Xue B, Han B, Li H, et al. Understanding ecological civilization in China: From political context to science. Ambio, 2023, 52(12): 1895-1909.https://doi.org/10.1007/s13280-023-01897-2

[7]Lu L, Su X, Hu S, et al. Green transition in manufacturing: Dynamics and simulation.PLOS ONE, 2023, 18(1): e0280389. https://doi.org/10.1371/journal.pone.0280389

[8]Guo M, Wang H, Kuai Y. Environmental regulation and green innovation: Evidence from heavily polluting firms in China. Finance research letters, 2023, 53: 103624.https://doi.org/10.1016/j.frl. 2022.103624

[9]Wang Q, Xu X, Liang K. The impact of environmental regulation on firm performance: Evidence from the Chinese cement industry. Journal of Environmental Management, 2021, 299: 113596. https://doi.org/10.1016/j.jenvman.2021.113596

[10]Bai C, Sarkis J, Dou Y. Corporate sustainability development in China: review and analysis. Industrial Management & Data Systems, 2015, 115(1): 5-40. https://doi.org/10.1108/IMDS-09-2014-0258

[11]	Zhou L, Li W, Teo B S X, et al. The effect of green transformation on the operating efficiency of green M&A enterprises: Evidence from China. The Journal of Asian Finance, Economics and Business, 2022, 9(1): 299-310. https://doi.org/10.13106/jafeb.2022.vol9.no1.0299

[12]Caiazza S, Galloppo G, Paimanova V. The role of sustainability performance after merger and acquisition deals in short and long-term. Journal of Cleaner Production, 2021, 314: 127982. https://doi.org/10.1016/j.jclepro.2021.127982

[13]	Andriuškevičius K, Štreimikienė D. Sustainability framework for assessment of mergers and acquisitions in energy sector. Energies, 2022, 15(13): 4557. https://doi.org/10.3390/en15134557

[14]	Leon-Gonzalez R, Tole L. The determinants of mergers & acquisitions in a resource-based industry: What role for environmental sustainability?. Review of Economic Analysis, 2015, 7(2): 111-134. https://doi.org/10.15353/rea.v7i2.1417

[15]	González-Torres T, Rodríguez-Sánchez J L, Pelechano-Barahona E, et al. A systematic review of research on sustainability in mergers and acquisitions. Sustainability, 2020, 12(2): 513. https:// doi.org/10.3390/su12020513

[16]Zhao X, Jia M. Sincerity or hypocrisy: can green M\&A achieve corporate environmental governance?. Environmental Science and Pollution Research, 2022: 1-13. https://doi.org/10.1007 /s11356-021-17464-9

[17]	Cranston R. Regulation and Deregulation: General Issues. University of New South Wales law journal, 1982, 5(1): 1.

[18]	McKean, Roland N. Enforcement Costs in Environmental and Safety Regulation. Policy Analysis, 1980, 6(3): 269. https://www.jstor.org/stable/42783602

[19]	Bulger M, Davison P. The Promises, Challenges, and Futures of Media Literacy. The journal of media literacy education, 2018, 10(1): 1-21. https://doi.org/10.23860/JMLE-2018-10-1-1

[20]	Silva M J, Caled D. Digital media and misinformation: An outlook on multidisciplinary strategies against manipulation. Computational Social Science. 2021: 1-37. https://doi.org/10.1007/s42001-021-00118-8

[21]	Khan A, Chenggang Y, Bano S, et al. The empirical relationship between environmental degradation, economic growth, and social well-being in Belt and Road Initiative countries. Environmental Science and Pollution Research, 2020, 27(24): 30800-30814. https://doi.org/10.1007 /s11356-020-09058-8

[22]Eisenmenger N, Pichler M, Krenmayr N, et al. The Sustainable Development Goals prioritize economic growth over sustainable resource use: a critical reflection on the SDGs from a socio-ecological perspective. Sustainability Science, 2020, 15(4): 1101-1110. https://doi.org/10.1007/ s11625-020-00813-x

[23]	Hundloe T. Sustainability Perspectives: Ecological, Economic and Social. Environmental Impact Assessment: Incorporating Sustainability Principles, 2021: 49-80. https://doi.org/10.1007/978-3-030-80942-3_3

[24]	Harsanyi J C. Games with Incomplete Information Played by Bayesian Players, I-III. Management Science, 2004, 50(12): 1804-1817. https://doi.org/10.1287/mnsc.14.3.159

[25]Simon H A. A Behavioral Model of Rational Choice. 1953. https://doi.org/10.2307/1884852

[26]	STREN P C. Toward a coherent theory of environmentally significant behaviour. Journal of Social Issues, 2000, 56(3): 407-24.

[27]	Ellerman A D. Pricing Carbon: The European Union Emissions Trading Scheme. 2010.

[28]	Hahn R W, Hird J A. The Costs and Benefits of Regulation: Review and Synthesis. Yale Journal on Regulation, 1991, 8(1): 9.

[29]	Zou L, Ma J. Does carbon emission trading scheme improve corporate green mergers and acquisitions? Evidence from Chinese industrial enterprises. Environment, Development and Sustainability, 2024. https://doi.org/10.1007/s10668-024-05327-5

[30]Qian J, Zhou Y. Can an emission trading policy promote green transformation of regional economies?: Evidence from China. Journal of Water and Climate Change, 2024, 15(1): 171-191. https://doi.org/10.2166/wcc.2023.540

[31]	Hertz N. The Silent Takeover: Global Capitalism and the Death of Democracy. 2001.

[32]	Schiffrin A. Media capture: How money, digital platforms, and governments control the news. 2021. https://doi.org/10.7312/schi18882-019

[33]	Geng Y, Chen J, Liu T, et al. Public environmental attention, media coverage, and corporate green innovation: evidence from heavily polluting industries in China. Environ Sci Pollut Res, 2023, 30(37): 86911-26. https://doi.org/10.1007/s11356-023-28369-0

[34]	Bu M, Liu X, Zhang B, et al. Governance of Corporate Greenwashing through ESG Assurance. Systems, 2024, 12(9): 365. https://doi.org/10.3390/systems12090365

[35] Liang X, Li S, Luo P, et al. Green mergers and acquisitions and green innovation: An empirical study on heavily polluting enterprises. Environ Sci Pollut Res, 2022, 29(32): 48937-52. https://doi.org/10.1007/s11356-022-19270-3

[36]Kolcava D. Greenwashing and public demand for government regulation. Journal of Public Policy, 2023, 43(1): 179-98. https://doi.org/10.1017/S0143814X22000277

[37]	Li, Liu Q, Tang D, et al. Media reporting, carbon information disclosure, and the cost of equity financing: evidence from China. Environ Sci Pollut Res, 2017, 24(10): 9447-59. https://doi.org/ 10.1007/s11356-017-8614-4

[38]	Mei Z, Zhang J, Zhou Q. The role of government in helping SMEs to access finance: An evolutionary game modeling and simulation approach. Plos One, 2024, 19(12): e0315941. https://doi.org/10.1371/journal.pone.0315941

[39]	Dubbeldam J, Brown J S, Stanková K, et al. Fisheries management as a Stackelberg Evolutionary Game: Finding an evolutionarily enlightened strategy. Plos One, 2021, 16(1): 1-20. https://doi.org/ 10.1371/journal.pone.0245255

[40]	Xu Y, Wang W, Gao H, et al. The Impact of Green Mergers and Acquisitions on Corporate Environmental Performance: Evidence from China’s Heavy-Polluting Industries. Sustainability-Basel, 2024, 16(9): 1-24. https://doi.org/10.3390/su16093796

[41] He S, Wei Y, Li W. Research on the impact of green mergers and acquisitions of heavily polluting enterprises on the quality of environmental information disclosure: empirical evidence from listed companies in China. Environment, Development and Sustainability, 2024. https://doi.org/10.1007 /s10668-024-04525-5

[42]Stefan A, Paul L. Does It Pay to Be Green? A Systematic Overview. Academy of Management Perspectives, 2008, 22(4): 45-62. https://www.jstor.org/stable/27747478

[43]	Zhai T. An empirical study of marine environmental civil public-interest litigation in China: Based on 216 cases from 2015 through 2022. Ocean and Coastal Management, 2024, 253: 107164. https://doi.org/10.1016/j.ocecoaman.2024.107164

[44]	Wang Y, Zhang J. Corporate Governance, Media Coverage, and Corporate Environmental Protection Investment: Empirical Evidence from Listed Companies in China’s High-Pollution Industries. Sustainability-Basel, 2023, 15(11): 8643. https://doi.org/10.3390/su15118643

[45]Sparks C, Wang H, Huang Y, et al. The impact of digital media on newspapers: Comparing responses in China and the United States. Global Media and China, 2016, 1(3): 186-207. https://doi.org/10.1177/2059436416666385

[46]Shearer E, Mitchell A. News use across social media platforms in 2020. 2021.

[47]Jiang Z, Liu B, Liu J, et al. Media Attention and Regulatory Efficiency of Corporate Violations: Evidence from China. Social Science Research Network, 2020. https://doi.org/10.1016/j.jaccpubpol.2021.106931

[48]	Gentzkow M, Allcott H. Social Media and Fake News in the 2016 Election. Research Papers in Economics, 2017. https://doi.org/10.1257/jep.31.2.211

[49]Linde C V D, Porter M E. Green and Competitive: Ending the Stalemate. Journal of Business Administration and Policy Analysis, 1995: 215. https://doi.org/10.1016/0024-6301(95)99997-E

[50]	Zhang S, Zhang C, Cai W, et al. The 2023 China report of the Lancet Countdown on health and climate change: taking stock for a thriving future. The Lancet Public Health, 2023, 8(12): e978-e95. https://doi.org/10.1016/S2468-2667(23)00245-1

[51]	Wang H. Pollution regulation and abatement efforts: evidence from China. Ecol Econ, 2002, 41(1): 85-94. https://doi.org/10.1016/S0921-8009(02)00016-2

[52]	Godlewska M, Pilewicz T. Entrepreneurial activities of local governments in their investment attractiveness context–evidence from Poland. Local Government Studies, 2022, 48(3): 590-614. https://doi.org/10.1080/03003930.2020.1842736

[53]	Zhang B, Cao C, Gu J, et al. A New Environmental Protection Law, Many Old Problems? Challenges to Environmental Governance in China. Journal of Environmental Law, 2016, 28(2): 325-35. https://doi.org/10.1093/jel/eqw014

[54]	Evaluation U of W I for H M and, Weltbank. The cost of air pollution: strengthening the economic case for action. 2016.

[55]	Niu Y, Chen R, Kan H. Air Pollution, Disease Burden, and Health Economic Loss in China. Advances in Experimental Medicine and Biology. Springer Singapore. 2017: 233-42. https://doi.org/10.1007/978-981-10-5657-4_10

[56]Zhang L, Mol A P J, He G. Transparency and information disclosure in China's environmental governance. Current Opinion in Environmental Sustainability, 2016, 18: 17-24. https://doi.org/10.1016/j.cosust.2015.03.009

[57]Sun D, Zeng S, Chen H, et al. Monitoring effect of transparency: How does government environmental disclosure facilitate corporate environmentalism?. Business Strategy and the Environment, 2019, 28(8): 1594-607. https://doi.org/10.1002/bse.2335

[58]	Bin O, Zheng Y, Jingsi C. Environmental awareness and environmental behavior of city residents in China. Economic Geography. 2015;35(11):179-83.https://doi.org/10.15957/j.cnki.jjdl.2015.11.025

[59]	Gray W, Zheng H R. General Principles of Civil Law of the People's Republic of China. American Journal of Comparative Law, 1986, 34(4): 715. https://doi.org/10.2307/840330

[60]	Wu W. The reform of the compensation system for ecological and environmental damage in China. Natural Resources Journal, 2020, 60(1): 63-102. https://www.jstor.org/stable/26912772

[61]	Xu L, Xie L. Environmental Public Interest Litigation in China: A Critical Examination. Transnational Environmental Law, 2021: 1-25. https://doi.org/10.1017/S2047102520000448

[62] Haws K L, Winterich K P, Naylor R W. Seeing the world through GREEN-tinted glasses: Green consumption values and responses to environmentally friendly products. Journal of Consumer Psychology, 2014, 24(3): 336-54. https://doi.org/10.1016/j.jcps.2013.11.002

[63]Romani S, Grappi S, Bagozzi R P. Corporate Socially Responsible Initiatives and Their Effects on Consumption of Green Products. Journal of Business Ethics, 2016, 135(2): 253-64. https://doi.org/10.1007/s10551-014-2485-0

[64]	Slovic P. Perception of risk. Science, 1987, 236(4799): 280-5. https://doi.org/10.1126/science.3563507
